# Compatibility Study of Peptide and Glycerol Using Chromatographic and Spectroscopic Techniques: Application to a Novel Antimicrobial Peptide Cbf-14 Gel

**DOI:** 10.3390/pharmaceutics15122784

**Published:** 2023-12-15

**Authors:** Jixue Yang, Yitong Huo, Xin Jin, Meiyun Liu, Yuting Lu, Lingman Ma, Changlin Zhou, Taijun Hang, Min Song

**Affiliations:** 1 Department of Pharmaceutical Analysis, China Pharmaceutical University, Nanjing 211198, China; 3221010221@stu.cpu.edu.cn (J.Y.); 3219010166@stu.cpu.edu.cn (Y.H.); 3221010469@stu.cpu.edu.cn (X.J.); 3319010364@stu.cpu.edu.cn (M.L.); luyt@cpu.edu.cn (Y.L.); hangtj@cpu.edu.cn (T.H.); 2 State Key Laboratory of Natural Medicines, School of Life Science and Technology, China Pharmaceutical University, Nanjing 211198, China; malingman1987@126.com (L.M.); cl_zhou@cpu.edu.cn (C.Z.)

**Keywords:** Cbf-14, peptide preparation, glycerol, aldehydes, Maillard reaction, compatibility study, 2D LC–QTOF-MS/MS

## Abstract

The interactions between active pharmaceutical ingredients (APIs) and excipients may lead to API degradation, thereby affecting the safety and efficacy of drug products. Cbf-14 is a synthetic peptide derived from Cathelicidin-BF, showing potential for bacterial and fungal infections. In order to assess impurities in Cbf-14 gel, we developed a two-dimensional liquid chromatography coupled with quadrupole/time-of-flight mass spectrometric method. A total of eleven peptide degradation impurities were identified and characterized. Furthermore, the compatibility tests were conducted to evaluate the interactions of Cbf-14 with glycerol and methylcellulose, respectively. The results revealed that the impurities originated from condensation reactions between Cbf-14 and aldehydes caused by glycerol degradation. Several aldehydes were employed to validate this hypothesis. The formation mechanisms were elucidated as Maillard reactions between primary amino groups of Cbf-14 and aldehydes derived from glycerol degradation. Additionally, the compatibility of Cbf-14 with glycerol from different sources and with varying storage times was investigated. Notably, the interaction products in the gel increased with extended storage time, even when fresh glycerol for injection was added. This study offers unique insights into the compatibility study of peptides and glycerol, contributing to the ongoing quality study of Cbf-14 gel. It also serves as a reference for the design of other peptide preparations and excipients selections.

## 1. Introduction

Since the discovery of penicillin in 1928, the world has entered a golden age of antibiotics. These powerful treatments for infections led to a significant reduction in infectious-related deaths. However, the excessive use of antibiotics has given rise to the emergence of multi-drug-resistant bacteria, posing a severe threat to public health [1,2]. In recent years, antimicrobial peptides (AMPs) and their analogs have garnered attention as promising alternatives to traditional antibiotics. This is owing to their broad-spectrum antimicrobial capabilities and a reduced likelihood of developing drug resistance [3].

**Figure 1:**
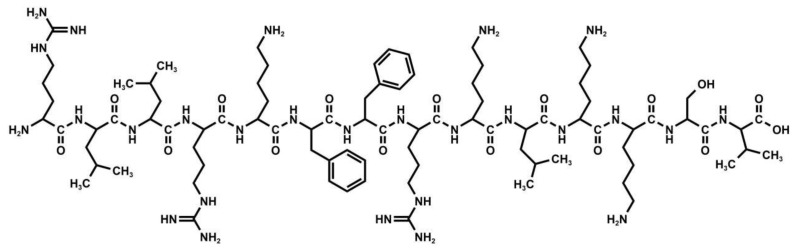
Chemical structure of Cbf-14.

Cbf-14 is a structurally modified peptide derived from Cathelicidin-BF (BF-30), consisting of 14 amino acids with the sequence of RLLRKFFRKLKKSV (Figure 1). Previous studies have suggested that Cbf-14 possesses both antibacterial and antifungal activities, highlighting its clinical potential for treating bacterial and fungal infections [4,5]. Cbf-14 gel is a novel topical preparation designed for local skin sterilization and post-operative wound anti-infection prevention. In this gel, Cbf-14 serves as an active pharmaceutical ingredient (API), glycerol functions as a humectant, and methylcellulose acts as a gel matrix. 

While biopharmaceuticals have shown remarkable therapeutic effects for few selected diseases, their widespread application is hindered by issues such as unstable structures, vulnerability to degradation, and stringent storage requirements. One critical aspect that deserves attention in this context is the interaction between APIs and excipients, as excipients can potentially interact with the API directly or indirectly. In some cases, they act as catalysts, hastening the degradation of the API. For instance, salmon calcitonin has been observed to undergo a temperature-dependent decrease when reacting with carbomer polymers [6], atrial natriuretic peptide can undergo acylation reaction when exposed to poly(lactide-co-glycolide) (PLGA) [7], and the presence of hyaluronic acid has been found to accelerate the degradation of daptomycin [8]. 

Recently, the U.S. Food and Drug Administration (FDA) released new guidance on Abbreviated New Drug Applications (ANDAs), underscoring the significance of quality control of synthetic peptide drug products [9]. In alignment with International Conference on Harmonization (ICH) guidelines [10], impurities exceeding 0.1% of the API necessitate identification and characterization as part of a risk assessment, concerning their impact on patient safety and product efficacy. Typically, pharmaceutical research involving peptides employs various technologies, including liquid chromatography coupled with high-resolution mass spectrometry (LC–HRMS) [11], capillary electrophoresis (CE) [12], nuclear magnetic resonance (NMR) spectroscopy [13], circular dichroism (CD) spectroscopy [14], X-ray diffraction (XRD) [15], etc. During the development of Cbf-14 gel, an unusual degradation of APIs following long-term storage was encountered. This degradation was not observed during the previous impurity profile study of the Cbf-14 drug substance [16], and the impurity levels exceeded the ICH identification threshold. Consequently, it is imperative to establish an appropriate analytical method to characterize these impurities and gain further insights into their origin. 

**Figure 2:**
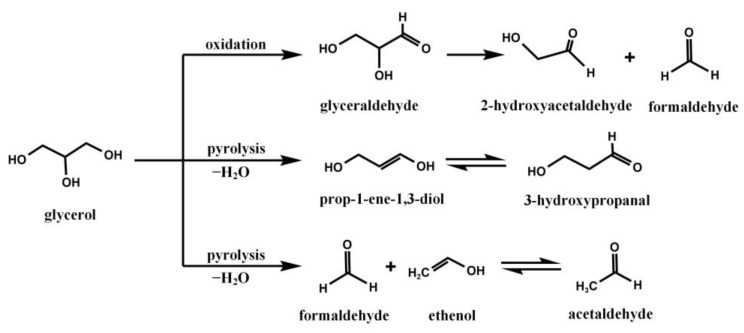
The degradation pathways of glycerol under certain conditions.

Glycerol is widely used in pharmaceutical preparations as a sweetening agent, co-solvent, humectant, or lubricant. More than 850 products, including gels, capsules, tablets, creams, syrups, and others, contain glycerol [17]. When stored appropriately, glycerol exhibits excellent stability. However, it is highly susceptible to degradation when subjected to repeated use [18]. A review of the literature reveals that glycerol degradation pathways can be categorized into anaerobic and aerobic processes. Under anaerobic conditions, glycerol primarily undergoes pyrolysis to yield formaldehyde, ethenol, and prop-1-ene-1,3-diol through various transition state mechanisms. These enols subsequently isomerize to generate acetaldehyde and 3-hydroxypropanal. In contrast, aerobic conditions may lead to the production of glyceraldehyde via glycerol oxidation, which further decomposes into formaldehyde and 2-hydroxyacetaldehyde [19,20]. The primary reaction equations are illustrated in Figure 2. These reactive impurities have the potential to induce physicochemical instability of the API. 

Therefore, we can legitimately infer that the potential stability changes in Cbf-14 most likely result from interactions between the API and excipients. Consequently, compatibility tests of Cbf-14 with glycerol and methylcellulose were further studied, respectively. Several aldehydes possibly present in glycerol were used to verify the impurity formation mechanisms. The primary degradation impurities were analyzed using reversed-phase (RP)-HPLC, two-dimensional liquid chromatography coupled to quadrupole/time-of-flight mass spectrometry (2D LC–QTOF-MS), or tandem mass spectrometry (MS/MS). The results indicated that Cbf-14 was incompatible with glycerol, even with fresh glycerol. All eleven new impurities originated from condensation reactions between the API and the reactive impurities present in glycerol. Additionally, the degradation trend of the API was analyzed. This is the first report of the compatible issues between a peptide and fresh glycerol. These findings highlight the general incompatibility between peptides and substances releasing aldehydes. Moreover, the discovery of susceptibility sites in Cbf-14 provides potential ideas for improving the drug stability from a structure modification perspective.

## 2. Materials and Methods

### 2.1. Chemicals and Reagents

Chemicals and reagents used for the preparation of samples and the mobile phases were as follows: Cbf-14 acetate freeze-dried powder (RLLRKFFRKLKKSV, batch number: C660QFK300-1/20110501-DG01; ≥99%) custom-synthesized by GenScript Biotech Corp. (Nanjing, China); glycerol from different suppliers: from Aladdin Reagent Co., Ltd. (Shanghai, China) (batch number: G2123136, L1920122; ≥99%), from Shanghai Lingfeng Chemical Reagent Co., Ltd. (Shanghai, China) (batch number: 20170711; ≥99%), from Sinopharm Chemical Reagent Co., Ltd. (Shanghai, China) (batch number: 20220121; ≥99%), from Guangzhou Jinhuada Chemical Reagent Co., Ltd. (Guangzhou, China) (batch number: 20120212; ≥99%); ethanolamine (≥99%), formic acid (≥98%), methylcellulose (40,000 mPa·s), phosphoric acid (85%-90%), and sodium sulfate anhydrous (≥99%) purchased from Aladdin Reagent Co., Ltd. (Shanghai, China); acetaldehyde (99.5%) and formaldehyde solution (37–40%) from Macklin Biochemical Co., Ltd. (Shanghai, China); dl-glyceraldehyde (90%) and 3-hydroxypropanal (95%) from Bide Pharmatech Co., Ltd. (Shanghai, China); HPLC-grade acetonitrile supplied by Merck (Darmstadt, Germany); and ultrapure water (18.25 MΩ cm^−1^) purified with a Milli Q-Plus purification system (Millipore, Bedford, MA, USA).

### 2.2. Sample Preparation

#### 2.2.1. Preparation of Cbf-14 Gel

**Scheme 1:**
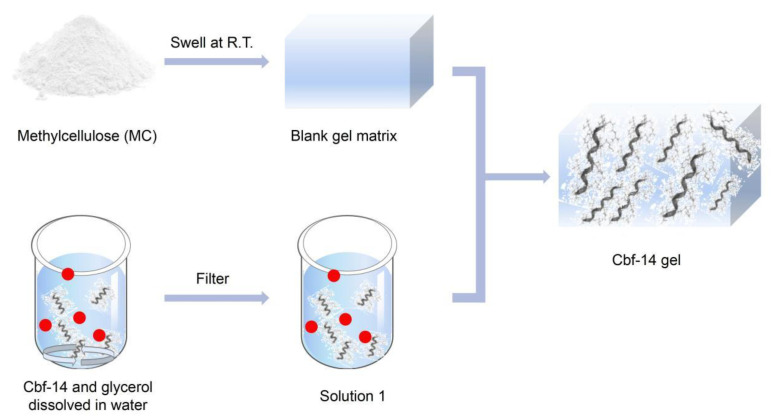
The procedure of Cbf-14 gel preparation.

The process of preparing Cbf-14 gel is illustrated in Scheme 1. In summary, 0.2 g of Cbf-14 acetate freeze-dried powder and 1 g of glycerol were mixed and dissolved in an appropriate amount of sterile distilled water at room temperature to form solution 1. Simultaneously, 0.2 g of methylcellulose was evenly swollen in 2 g of water at room temperature for 6 h to obtain blank matrix. Finally, solution 1 was combined with the blank matrix after being filtered through a 0.22 μm filter. Water was added to achieve a final gel weight of 10 g, and the mixture was stirred until it reached a homogenous consistency.

For Cbf-14 formulations with either glycerol or methylcellulose as the excipient additive, the preparation methods were nearly identical to the aforementioned process, with the only exception being the use of an equal amount of sterile distilled water as a substitute for the omitted ingredient.

The formulation sample solutions of Cbf-14 were prepared at a concentration of 1 mg/mL in water.

#### 2.2.2. Synthesis of the Peptide-Condensed Impurities

A total of 1 mg of freeze-dried Cbf-14 acetate powder was dissolved in 100 μL of a 0.02% (*w*/*w*) formaldehyde aqueous solution, a 0.01% (*v*/*v*) acetaldehyde aqueous solution, a 0.1 mg/mL glyceraldehyde aqueous solution, and a 0.005 mg/mL 3-hydroxypropanal aqueous solution, respectively. The solutions stood at room temperature for 30 min. Finally, ultrapure water was added to the reaction mixtures to stop the reaction and dilute Cbf-14 to a concentration of 1 mg/mL.

### 2.3. Equipment and Chromatographic Conditions

The Shimadzu online trap-free Nexera-XR column-switching system, coupled with LC–MS-9030 quadrupole/time-of-flight mass spectrometer (Shimadzu, Tokyo, Japan), was employed for the separation, quantitation, and characterization of all impurities. The LC effluent was introduced into the electrospray ionization (ESI) source inlet without splitting.

#### 2.3.1. Chromatographic Conditions

A two-dimensional system was established by combining two RP-HPLC systems. In the first RP-HPLC system, chromatographic separation was achieved at 40 °C on a Shimadzu VP-ODS column (250 mm × 4.6 mm, 5 μm particle size). The mobile phases consisted of 200 mM sodium sulfate buffer (dissolve 28.4 g of sodium sulfate anhydrous in 1 L of water, add 2.7 mL of phosphoric acid, and adjust to pH 2.5 with ethanolamine) in acetonitrile (82:18, *v*/*v*) for mobile phase A and 50% acetonitrile for mobile phase B. The gradient program was as follows: 0–10 min, linear gradient 10% to 20% B; 10–50 min, linear gradient 20% to 48% B; 50–52 min, linear gradient 48% to 10% B; 52–60 min, an isocratic 10% B. The flow rate was 1.0 mL/min, and the injection volume was 20 μL. Analytes were monitored using a PDA detector within the wavelength range of 190–400 nm.

The effluent desalting treatment in the second RP-HPLC system was carried out at 40 °C using a Teknokroma Partisil column (4.6 mm × 100 mm, 5 μm). Eluent A consisted of a 0.1% formic acid solution in acetonitrile (95:5, *v*/*v*), while eluent B was a mixture of a 0.1% formic acid solution and acetonitrile (40:60, *v*/*v*). The gradient program was configured as follows: 0–10 min, a linear gradient 0% to 60% B; 10–10.1 min, a linear gradient 60% to 0% B; 10.1–14 min, isocratic elution with 0% B. The flow rate was maintained at 0.5 mL/min, and the UV detection was performed at a wavelength of 220 nm.

#### 2.3.2. Mass Spectrometry Conditions

Mass spectrometric analysis was conducted in positive ESI mode. Nitrogen was used as nebulizer gas at a rate of 3.0 L/min. Both the drying gas and heating gas were set to flow rate at 10.0 L/min. The desolvation line’s temperature was maintained at 250 °C, and the heat block was held at 400 °C. The detector voltage was set at 1.94 kv, and the full scan range covered the range of 100–2000 *m*/*z*. For MS/MS experiments, argon gas was employed as collision gas, with the collision energy ranging from 5 to 50 eV.

All data acquisition and analysis of mass spectrometry were performed using Shimadzu LabSolutions software 5.118. Theoretical MS/MS fragment ions, calculated based on molecular weights, were determined using LabSolutions Insight software 3.7.

## 3. Results and Discussion

### 3.1. Two-Dimensional RP-HPLC Method Development

**Figure 3:**
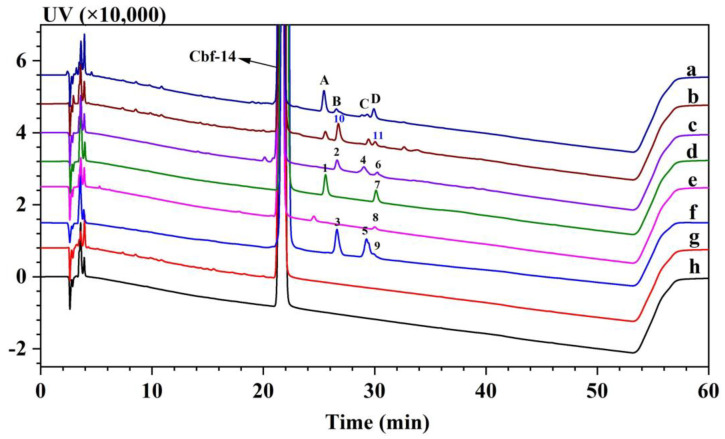
RP-HPLC chromatograms of Cbf-14 in different formulations and condensed impurities with aldehydes. (a) Cbf-14 gel (with both glycerol and methylcellulose as excipients) stored at 4 °C for 2 months; (b) formulation with Cbf-14 and glycerol alone stored at 4 °C for 2 months; (c) Cbf-14-condensed impurities with formaldehyde; (d) Cbf-14-condensed impurities with acetaldehyde; (e) Cbf-14-condensed impurities with 3-hydroxypropanal; (f) Cbf-14-condensed impurities with glyceraldehyde; (g) formulation with Cbf-14 and methylcellulose alone stored at 4 °C for 2 months; and (h) fresh-prepared Cbf-14 gel (with both glycerol and methylcellulose as excipients) (PDA extract at 220 nm). A–D: The peptide related substances detected in the gel.

Chromatography coupled with mass spectrometry is a powerful technique for profiling impurities. However, when following pharmacopeias, the most commonly used chromatographic separation for peptides relies on RP-LC with an acetonitrile gradient, supplemented by buffers such as sulphate, phosphate, triethylamine, and trifluoroacetic acid in the mobile phase (Table 1). These mobile phase additives are effective in separating structurally related substances but are not amenable to mass spectrometry due to their non-volatility or their tendency to cause ion suppression. The redevelopment of alternative, volatile elution agents is an expensive and time-consuming process. More importantly, this process can result in changes in chromatographic retention and resolution, despite maintaining a constant pH [21]. To address this challenge, a practical 2D LC method was developed to perform online desalting before MS detection. This approach avoids altering the original non-volatile HPLC method and the retention times of the impurities. Sulphate buffer and acetonitrile were employed as additives to minimize baseline noise caused by the terminal adsorption of the mobile phase at low ultraviolet detection wavelength, resulting in improved sensitivity and peak shapes. Following the developed chromatographic method, Cbf-14 exhibited a retention time of 22 min. A range of impurities, labeled as peak A to D, were detected between 25 min and 30 min (Figure 3). Peak resolution was mostly ≥1.5, achieving effective separation for identifying these impurities.

### 3.2. Analysis of the Source of Peptide Degradation Impurities

To trace the source of degradation peptide impurities in the gel, various formulations of Cbf-14, each containing either glycerol or methylcellulose as excipient additive, were prepared. Based on the chromatograms shown in Figure 3, the unusual degradation was observed when Cbf-14 was combined with glycerol, in stark contrast to the absence issues when methylcellulose was used.

Comparison of Figure 3(a,b) revealed that the gel formulation containing both glycerol and methylcellulose as excipients exhibited better stability when contrasted with the formulation containing glycerol alone as an additive. The oxidized impurities at the N-terminal of Cbf-14 [16], with contents of 0.314% and 0.336% (0.1% main component as self-compare), respectively, were not detected in the gel containing both excipient additives. To analyze the reasons, we believe that the addition of methylcellulose, which can increase the molecular density and viscosity of aqueous solutions, serves to inhibit peptide aggregation [26], thereby inhibiting peptide oxidation and hydrolysis, ultimately enhancing peptide stability in the solution. Consequently, it was confirmed that the introduction of glycerol led to the degradation peptide impurities, highlighting an incompatibility between the peptide and glycerol.

While glycerol has been widely used in pharmaceutical formulations, instances of glycerol causing stability or efficacy changes in the API were not rare. As reported in the previous literature, the presence of glycerol in beclomethasone aerosol particles may reduce the dissolution rate of particles and subsequently alter the pharmacokinetics of the drug [27]. Additionally, chemical instabilities have been observed in captopril [28], cetirizine [29], and insulin [18] when glycerol was present.

### 3.3. Characterization of the Impurities via 2D LC–QTOF-MS/MS

The peptide degradation impurities were characterized using 2D LC–QTOF-MS/MS. Based on the chromatographic and mass spectrometric data, a total of eleven peptide degradation impurities were identified and characterized. These impurities were observed as four peaks, labeled as peaks A, B, C, and D, in the chromatogram of the Cbf-14 gel (Figure 3(a)) due to the similar retention characteristics of the isomers.

**Figure 4:**
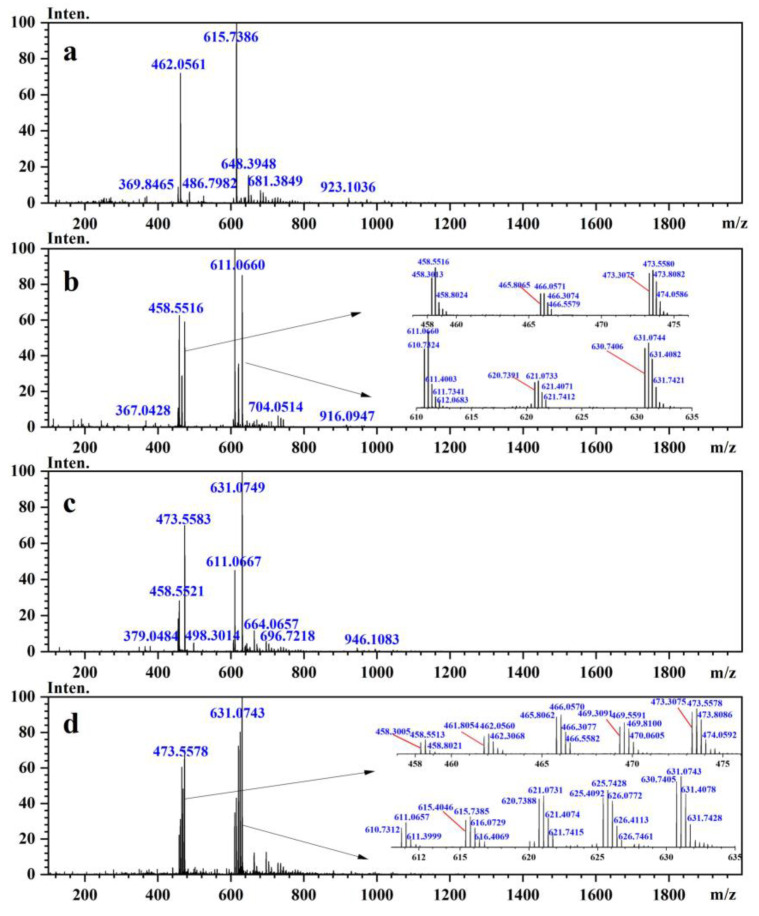
The mass spectra of the peptide-related impurities in Cbf-14 gel stored at 4 °C for 2 months. (**a**) Peak A; (**b**) peak B; (**c**) peak C; and (**d**) peak D.

In the positive ESI-QTOF-MS spectra of these impurities, quasi-molecular ions of [M + 3H]^3+^ at *m*/*z* 611.0660, 615.7386, 621.0733, 625.7428, and 631.0749 were observed, as depicted in Figure 4. These eleven detected impurities, labeled as impurities 1 to 11 (Figure 3), were categorized into five groups, as summarized in Table 2. Combined with the secondary mass spectra shown in Figure S1, it was speculated that Maillard reactions occurred between Cbf-14 and aldehydes, relying on primary amines. Cbf-14 contains eight primary amines in each molecule: three are associated with arginine guanidine groups, four are derived from lysine amino groups, and one is a free primary amine at the N-terminal. Given that the electron cloud of guanidine groups is evenly distributed, primary amines in guanidine groups exhibit lower reactivity compared to those at the N-terminal and lysine residues [30]. As a result, aldehydes were more likely to react with the primary amines at the N-terminal and lysine residues of the peptide rather than guanidine groups. This led to the formation of N-methylene-Cbf-14, N-ethylidene-Cbf-14, N-(2-hydroxyethylidene)-Cbf-14, N-(3-hydroxypropylidene)-Cbf-14, and N-(2,3-dihydroxypropylidene)-Cbf-14, respectively.

### 3.4. Analyzing the Mechanisms of Peptide Degradation Impurity Formation

As mentioned earlier, glycerol can be considered as a precursor of aldehydes under certain conditions. Aldehydes resulting from the cleavage of glycerol can react with primary amines to form imines. Therefore, we hypothesized that the peptide impurities were generated through chemical interactions between the peptide and the pyrolyzed products of glycerol. To confirm this hypothesis, we separately subjected Cbf-14 to formaldehyde, acetaldehyde, 3-hydroxypropanal, and glyceraldehyde, which are potential components found in glycerol. The interaction products were labeled with numbers from one to nine, as illustrated in Figure 3(c–f), according to their elution order. Unfortunately, impurities 10 and 11, containing in peaks B and D in Figure 3(a), respectively, could not be individually prepared due to the high reactivity of the initial aldehyde, 2-hydroxyacetaldehyde, making isolation challenging. The comparative results revealed that the impurities formed through these interactions matched the impurities detected in Cbf-14 gel (Figure 3).

**Figure 5:**
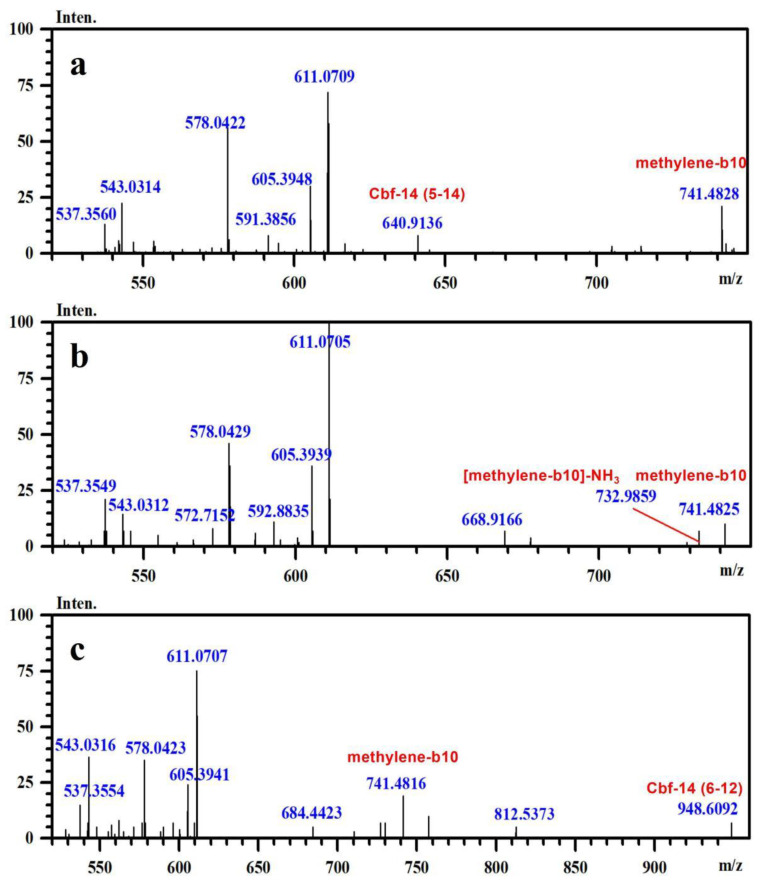
The secondary mass spectra of (**a**) impurity 2 (*m*/*z* 611.0709), (**b**) impurity 4 (*m*/*z* 611.0705), and (**c**) impurity 6 (*m*/*z* 611.0707).

In conjunction with the secondary mass spectra, it was determined that the susceptibility sites within the Cbf-14 amino acid sequence were primarily located at the N-terminal, along with lysine residues at positions K_5_ and K_9_. For example, taking N-methylene-Cbf-14, even though the proton ions displayed identical *m*/*z* values ranging from 611.0705 to 611.0709 for impurities two, four, and six, the alkylidene sites in the Cbf-14 amino acid could be determined using secondary mass spectrometry. As shown in Figure 5, impurity two was identified as methylene-Cbf-14 at N-terminal due to the presence of a product ion at *m*/*z* 640.9136 (Cbf-14 (5–14)). Impurity four was recognized as methylene-Cbf-14 at K_9_, as evidenced by product ions at *m*/*z* 741.4825 (methylene-b10) and *m*/*z* 732.9859 ([methylene-b10]-NH_3_), while lacking product ions at *m*/*z* 640.9136 (Cbf-14 (5–14)) or *m*/*z* 948.6092 (Cbf-14 (6–12)). Impurity six was identified as methylene-Cbf-14 at K_5_, supported by the presence of product ions at *m*/*z* 741.4816 (methylene-b10) and *m*/*z* 948.6092 (Cbf-14 (6–12)) but the absence of a product ion at *m*/*z* 640.9136 (Cbf-14 (5–14)). Regarding N-(2-hydroxyethylidene)-Cbf-14, impurities 10 and 11 were predicted based on the primary and secondary mass spectra information displayed in Figure 4b,d and electronic supplementary material (ESM, Figure S1g,h), respectively. All other product ion spectra are provided in Figure S1, and the fragmentation pathways of the impurities are extensively elucidated in Figure S2.

The results obtained indicated that Maillard reactions occurred when Cbf-14 was exposed to aldehydes, resulting in the production of degrading peptide impurities. Therefore, it was demonstrated that the aldehydes derived from the degradation of glycerol led to the chemical instability of Cbf-14.

The Maillard reaction, involving the covalent bonding of amino and carbonyl groups, plays a crucial role in food processing, contributing to its distinctive flavor [31]. However, in pharmaceutical formulations, this chemical reaction can pose a threat to the chemical stability of APIs, potentially impacting drug safety and efficacy. To prevent such degradation, several measures can be taken, including reducing temperature and pH [32], minimizing exposure to oxygen [33], and controlling water activity [34]. Most importantly, it is imperative to maintain an environment free from aldehydes. This entails not only preventing direct exposure to substances containing aldehydes but also avoiding potential sources of aldehydes, such as glycerol, polyethylene glycols [35], N,N-dimethylacetamide [36], polyethylene oxide, crospovidone [37], and others.

**Figure 6:**
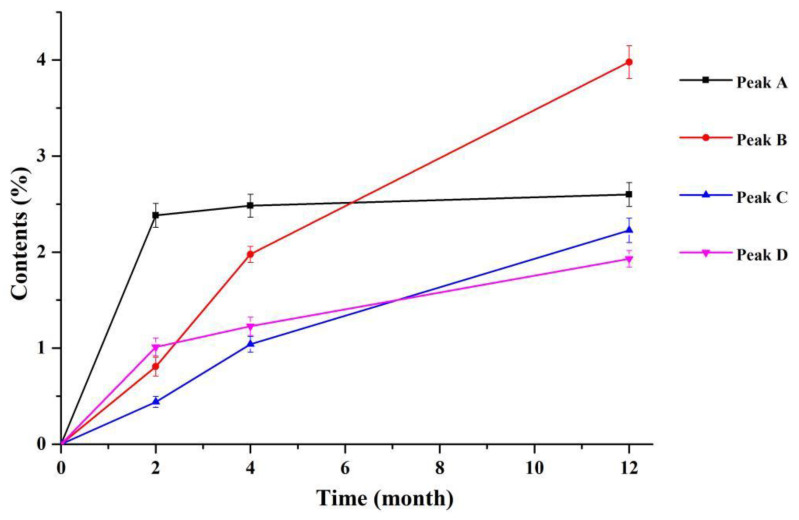
Contents of the impurities in Cbf-14 gels with different storage times via RP-HPLC. (Peak A contained impurity 1; peak B contained impurities 2, 3, and 10; peak C contained impurities 4 and 5; and peak D contained impurities 6, 7, 8, 9, and 11. Take 0.1% main component as self-compare; mean ± SD, *n* = 3).

In our current study, we demonstrated that chemical reactions can initiate between Cbf-14 and glycerol even at storage temperatures as low as 4 °C and even when fresh glycerol for injection was added. The degradation trend of Cbf-14 in the gel, as depicted in Figure 6, indicated that the content of peptide impurities increased over storage time, with no degradation detectable in freshly prepared gel.

## 4. Conclusions

In this study, we employed a practical 2D LC–QTOF-MS/MS method to investigate the compatibility between the novel antimicrobial peptide Cbf-14 and freshly prepared glycerol in a gel formulation. This research addresses a pressing need in quality control viewpoint, as it aims to characterize the impurities detected in the peptide gel. The results revealed that chemical interactions occurred when Cbf-14 was exposed to glycerol, even at a low temperature of 4 °C. Furthermore, the degradation of APIs progressively increased with extended storage time. It was confirmed that the impurities resulting from these interactions were serial products of Maillard reactions between primary amino groups of Cbf-14 and glycerol-derived substances. These findings highlight common degradation characteristics of Cbf-14 in gel, emphasizing the peptide’s high susceptibility to aldehyde conditions. Glycerol is a widely used excipient in biopharmaceutical formulations, but it is important to note that the quality control standards for glycerol can vary among pharmacopeias, especially in terms of reactive reducing substances like aldehydes and glucose. Therefore, both manufacturers and users should be vigilant about excipient quality control to ensure long-term compatibility between APIs and excipients. Additionally, the identification of susceptibility sites offers insights into enhancing the chemical stability of Cbf-14 through structure modifications.

## Figures and Tables

**Table 1:** The chromatographic conditions for the related substances in peptide products in major pharmacopeias.

Peptide	Ph.	Column	Mobile Phase	Flow Rate	Column Temperature	Detector	Acceptance Criteria	Reference
Teriparatide	USP 42	ODS (150 × 4.6 mm, 3.5 μm)	0.2 M sulfate buffer (pH 2.3) and acetonitrile (90:10) as mobile phase A, 0.2 M sulfate buffer (pH 2.3) and acetonitrile (50:50) as mobile phase B, gradient elution.	1.0 mL/min	40 °C	UV (214 nm)	Individual impurities: NMT 0.5%; sum of methionyl sulfoxides of teriparatide: NMT 0.5%; total impurities: NMT 2.5%.	[22]
	EP 10.0	ODS (150 × 4.6 mm, 3.5 μm)	0.2 M sulfate buffer (pH 2.3) and acetonitrile (90:10) as mobile phase A, 0.2 M sulfate buffer (pH 2.3) and acetonitrile (50:50) as mobile phase B, gradient elution.	1.0 mL/min	40 °C	UV (214 nm)	Individual impurities: NMT 0.5%; sum of impurities A, B, and C: NMT 0.5%; total impurities: NMT 2.0%.	[23]
Oxytocin	USP 42	ODS (120 × 4.6 mm, 5 μm)	0.1 M monobasic sodium phosphate as mobile phase A, acetonitrile and water (50:50) as mobile phase B, gradient elution.	1.5 mL/min	R.T.	UV (220 nm)	Total impurities: NMT 5.0% of the area of the oxytocin peak.	[22]
	ChP 2020	ODS	0.1 M monobasic sodium phosphate as mobile phase A, acetonitrile and water (50:50) as mobile phase B, gradient elution.	1.0 mL/min	_	UV (220 nm)	Individual impurities: NMT 2.0% of the area of the oxytocin peak; total impurities: NMT 5.0%.	[24]
	EP 10.0	ODS (125 × 4.6 mm, 5 μm)	0.1 M monobasic sodium phosphate as mobile phase A, acetonitrile and water (50:50) as mobile phase B, gradient elution.	1.0 mL/min	_	UV (220 nm)	Individual impurities: NMT 1.5% of the area of the oxytocin peak; total impurities: NMT 5.0%.	[23]
Leuprorelin	EP 10.0	ODS (100 × 4.6 mm, 3 μm)	0.15 M triethylamine buffer (pH 3.0), propanol, and acetonitrile (85:6:9), isocratic elution.	1.0~1.5 mL/min	_	UV (220 nm)	Impurity D: NMT 1.0%; individual impurities: NMT 0.5%, excluding impurity D; total impurities: NMT 2.5%.	[23]
	JP 18	ODS (100 × 4.6 mm, 3 μm)	0.15 M triethylamine buffer (pH 3.0), propanol, and acetonitrile (85:6:9), isocratic elution.	1.0~1.5 mL/min	25 °C	UV (220 nm)	Individual impurities: NMT 0.5%; total impurities: NMT 2.0%.	[25]
Octreotide Acetate	USP 42	C12 (250 × 4.6 mm, 4 μm)	0.02% of trifluoroacetic acid as mobile phase A, acetonitrile as mobile phase B, gradient elution.	1.0 mL/min	40 °C	UV (220 nm)	Individual impurities: NMT 0.5%; total impurities: NMT 2.0%.	[22]
	ChP 2020	ODS	0.22% tetramethylammonium hydroxide buffer (pH 5.4) and acetonitrile (90:10) as mobile phase A, 0.5% tetramethylammonium hydroxide buffer (pH 5.4) and acetonitrile (40:60) as mobile phase B, gradient elution.	_	_	UV (210 nm)	Individual impurities: NMT 1.0%; total impurities: NMT 2.0%.	[24]
	EP 10.0	ODS (250 × 4.6 mm, 5 μm)	0.028 M tetramethylammonium hydroxide buffer (pH 2.0) and acetonitrile (90:10) as mobile phase A, 0.028 M tetramethylammonium hydroxide buffer (pH 2.0) and acetonitrile (40:60) as mobile phase B, gradient elution.	1.8 mL/min	40 °C	UV (210 nm)	Individual impurities: NMT 0.5%; total impurities: NMT 2.0%.	[23]

**Table 2:** The structures of the related peptide impurities in Cbf-14 gel identified using 2D LC–QTOF-MS.

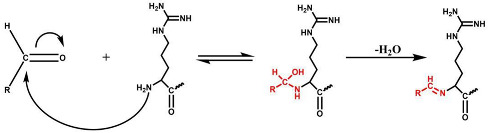							
Category No.	Substrate	Abbreviated Name	Predicted Formula (M)	Molecular Weight (Da)	Chemical Structure	Representive Impuritiy	Other Impurity Isomers
1	R = H	N-methylene -Cbf-14	C_87_H_151_N_27_O_16_	1830.1832	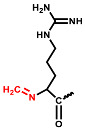	Impurity 2	Impurities 4 and 6
2	R = CH_3_	N-ethylidene -Cbf-14	C_88_H_153_N_27_O_16_	1844.1989	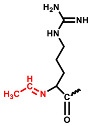	Impurity 1	Impurity 7
3	R = CH_2_OH	N-(2-hydroxy- -ethylidene) -Cbf-14	C_88_H_153_N_27_O_17_	1860.1938	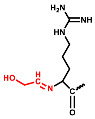	Impurity 10	Impurity 11
4	R = CH_2_CH_2_OH	N-(3-hydroxy- -propylidene) -Cbf-14	C_89_H_155_N_27_O_17_	1874.2094	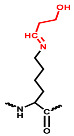	Impurity 8	/
5	R = CH(OH)CH_2_OH	N-(2,3- dihydroxy- -propylidene) -Cbf-14	C_89_H_155_N_27_O_18_	1890.2043	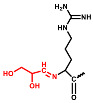	Impurity 3	Impurities 5 and 9

The red marks present the aldehyde groups modified on Cbf-14 amino acid residues.

## Data Availability

The data presented in the study are available on request from the corresponding author.

## Supplementary Materials

The following supporting information can be downloaded at: https://www.mdpi.com/article/10.3390/pharmaceutics15122784/s1, Figure S1: The secondary mass spectra of (a) impurity 1, *m*/*z* 615.7393; (b) impurity 3, *m/z* 631.0739; (c) impurity 5, *m*/*z* 631.0743; (d) impurity 7, *m*/*z* 615.7392; (e) impurity 8, *m*/*z* 625.7416; (f) impurity 9, *m*/*z* 631.0746; (g) impurity 10, *m/z* 621.0717; (h) impurity 11, *m*/*z* 621.0708; Figure S2. Plausible fragmentation pathways of (a) N-methylene-Cbf-14 (represented as impurity 2, and impurity 4, 6 were similar); (b) N-ethylidene-Cbf-14 (represented as impurity 1, and impurity 7 was similar); (c) N-(2-hydroxyethylidene)-Cbf-14 (represented as impurity 10, and impurity 11 was similar); (d) N-(3-hydroxypropylidene)-Cbf-14 (impurity 8); (e) N-(2,3-dihydroxypropylidene)-Cbf-14 (represented as impurity 3, and impurity 5, 9 were similar); Figure S3. Deduced structures of compounds in this study. (a) Cbf-14; (b) N-methylene-Cbf-14 (impurity 2, 4, 6); (c) N-ethylidene-Cbf-14 (impurity 1, 7); (d) N-(2-hydroxyethylidene)-Cbf-14 (impurity 10, 11); (e) N-(3-hydroxypropylidene)-Cbf-14 (impurity 8); (f) N-(2,3-dihydroxypropylidene)-Cbf-14 (impurity 3, 5, 9).
