# Supplementary material for: Compatibility Study of Peptide and Glycerol Using Chromatographic and Spectroscopic Techniques: Application to a Novel Antimicrobial Peptide Cbf-14 Gel
Source: Pharmaceutics. 2023 Dec 15;15(12):2784. doi: 10.3390/pharmaceutics15122784 (PMC10747037; doi:10.3390/pharmaceutics15122784)
Supplement: Supplementary file 1 [file pharmaceutics-15-02784-s001.zip › pharmaceutics-2731635-supplementary.pdf]

Electronic Supplementary Material for

Compatibility Study of Peptide and Glycerol Using Chromatographic and Spectroscopic Techniques: Application to a Novel Antimicrobial Peptide Cbf-14 Gel

Jixue Yang [1], Yitong Huo [1], Xin Jin [1], Meiyun Liu [1], Yuting Lu [1], Lingman Ma [2], Changlin Zhou [2], Taijun Hang [1] and Min Song [1,*]

[1] Department of Pharmaceutical Analysis, China Pharmaceutical University, Nanjing 211198, China; 3221010221@stu.cpu.edu.cn (J.Y.); 3219010166@stu.cpu.edu.cn (Y.H.); 3221010469@stu.cpu.edu.cn (X.J.); 3319010364@stu.cpu.edu.cn (M.L.); luyt@cpu.edu.cn (Y.L.); hangtj@cpu.edu.cn (T.H.)

[2] State Key Laboratory of Natural Medicines, School of Life Science and Technology, China Pharmaceutical University, Nanjing 211198, China; malingman1987@126.com (L.M.); cl_zhou@cpu.edu.cn (C.Z.)

* Correspondence: songmin@cpu.edu.cn; Tel.: +86-1358-40-52217

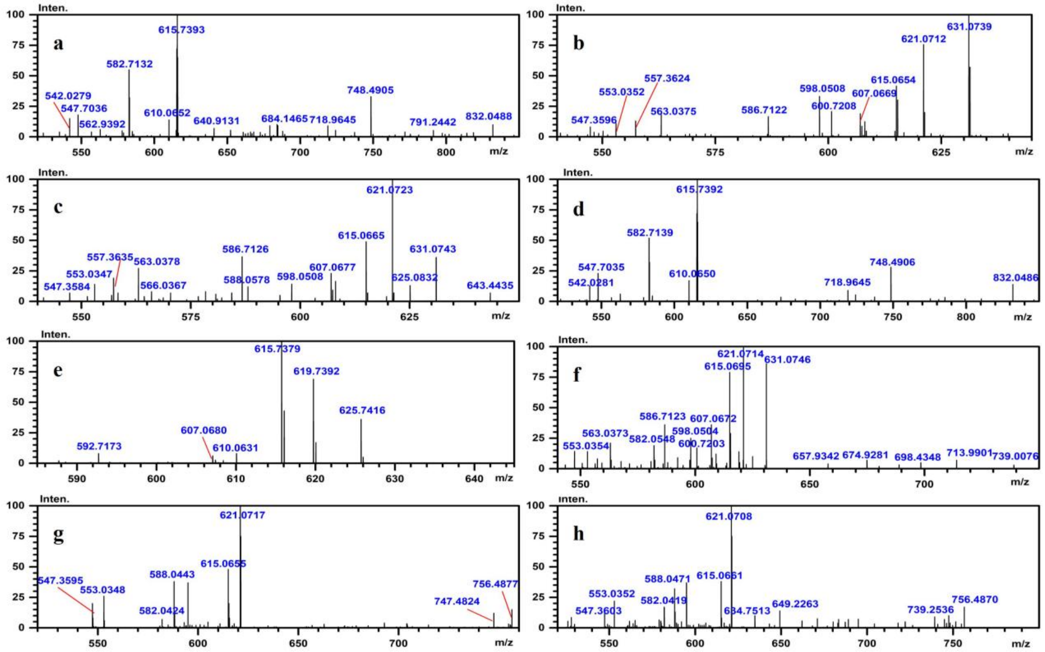

Figure S1. The secondary mass spectra of (a) impurity 1, *m/z* 615.7393; (b) impurity 3, *m/z* 631.0739; (c) impurity 5, *m/z* 631.0743; (d) impurity 7, *m/z* 615.7392; (e) impurity 8, *m/z* 625.7416; (f) impurity 9, *m/z* 631.0746; (g) impurity 10, *m/z* 621.0717; (h) impurity 11, *m/z* 621.0708.

a

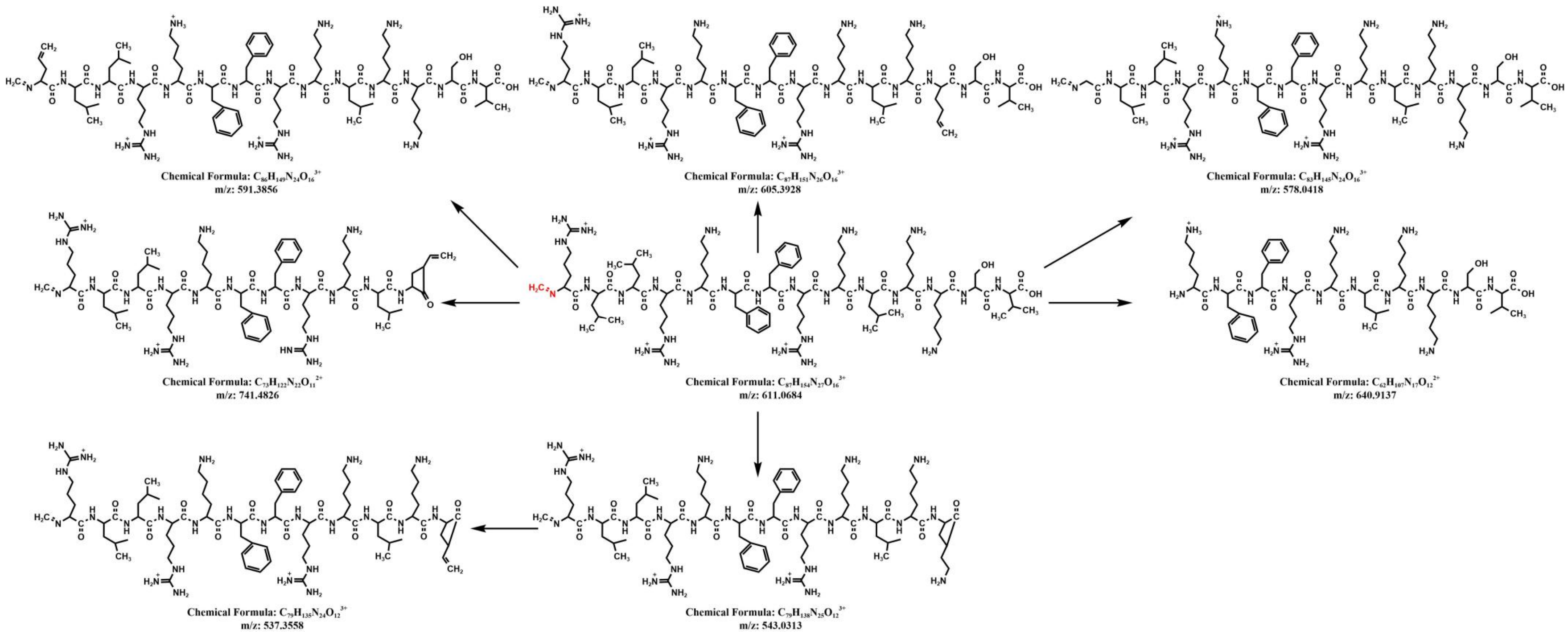
Chemical Formula: C86H149N24O16 3+
m/z: 591.3856
Chemical Formula: C87H151N26O16 3+
m/z: 605.3928
Chemical Formula: C83H145N24O16 3+
m/z: 578.0418
Chemical Formula: C73H122N22O11 2+
m/z: 741.4826
Chemical Formula: C87H154N27O16 3+
m/z: 611.0684
Chemical Formula: C62H107N17O12 2+
m/z: 640.9137
Chemical Formula: C79H135N24O12 3+
m/z: 537.3558
Chemical Formula: C79H138N25O12 3+
m/z: 543.0313

.

b

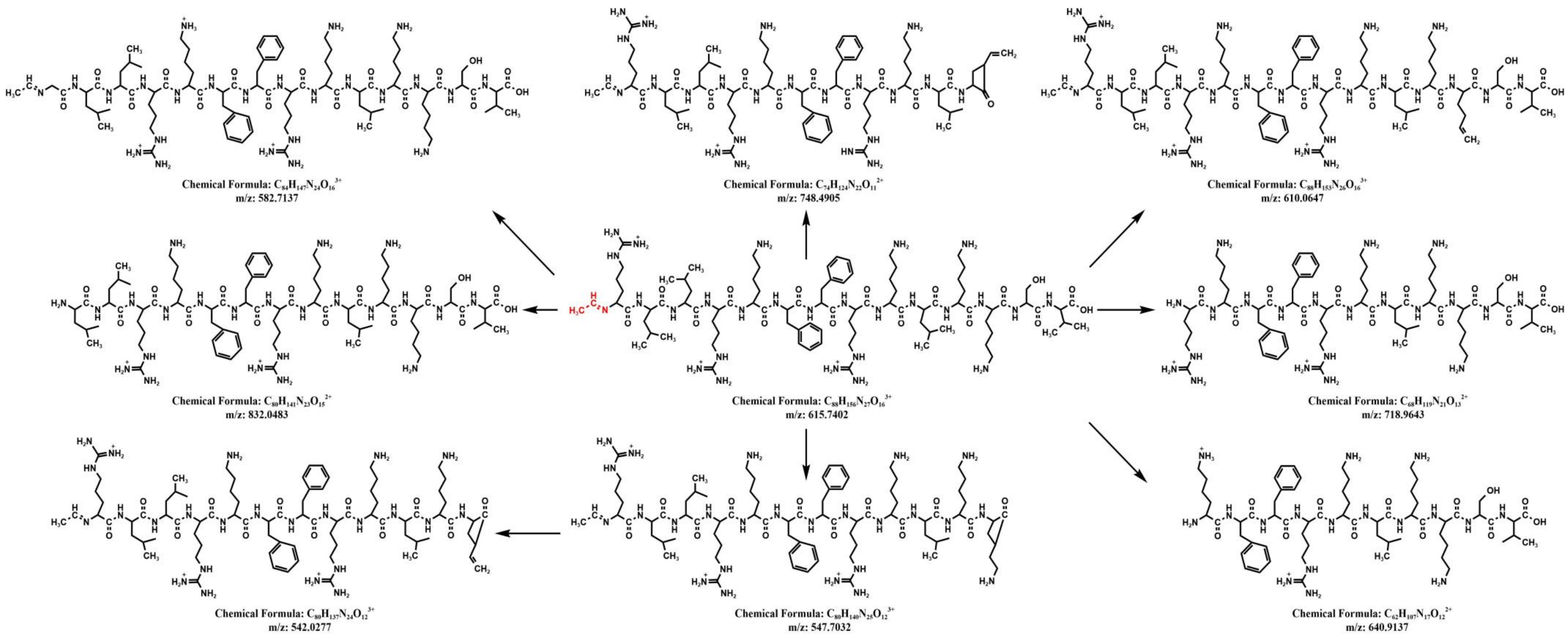

Chemical Formula: $C_{84}H_{147}N_{24}O_{16}{}^{3+}$
m/z: 582.7137
Chemical Formula: $C_{74}H_{124}N_{22}O_{11}{}^{2+}$
m/z: 748.4905
Chemical Formula: $C_{88}H_{153}N_{26}O_{16}{}^{3+}$
m/z: 610.0647
Chemical Formula: $C_{80}H_{141}N_{23}O_{15}{}^{2+}$
m/z: 832.0483
Chemical Formula: $C_{88}H_{156}N_{27}O_{16}{}^{3+}$
m/z: 615.7402
Chemical Formula: $C_{68}H_{119}N_{21}O_{13}{}^{2+}$
m/z: 718.9643
Chemical Formula: $C_{80}H_{137}N_{24}O_{12}{}^{3+}$
m/z: 542.0277
Chemical Formula: $C_{80}H_{140}N_{25}O_{12}{}^{3+}$
m/z: 547.7032
Chemical Formula: $C_{62}H_{107}N_{17}O_{12}{}^{2+}$
m/z: 640.9137

c

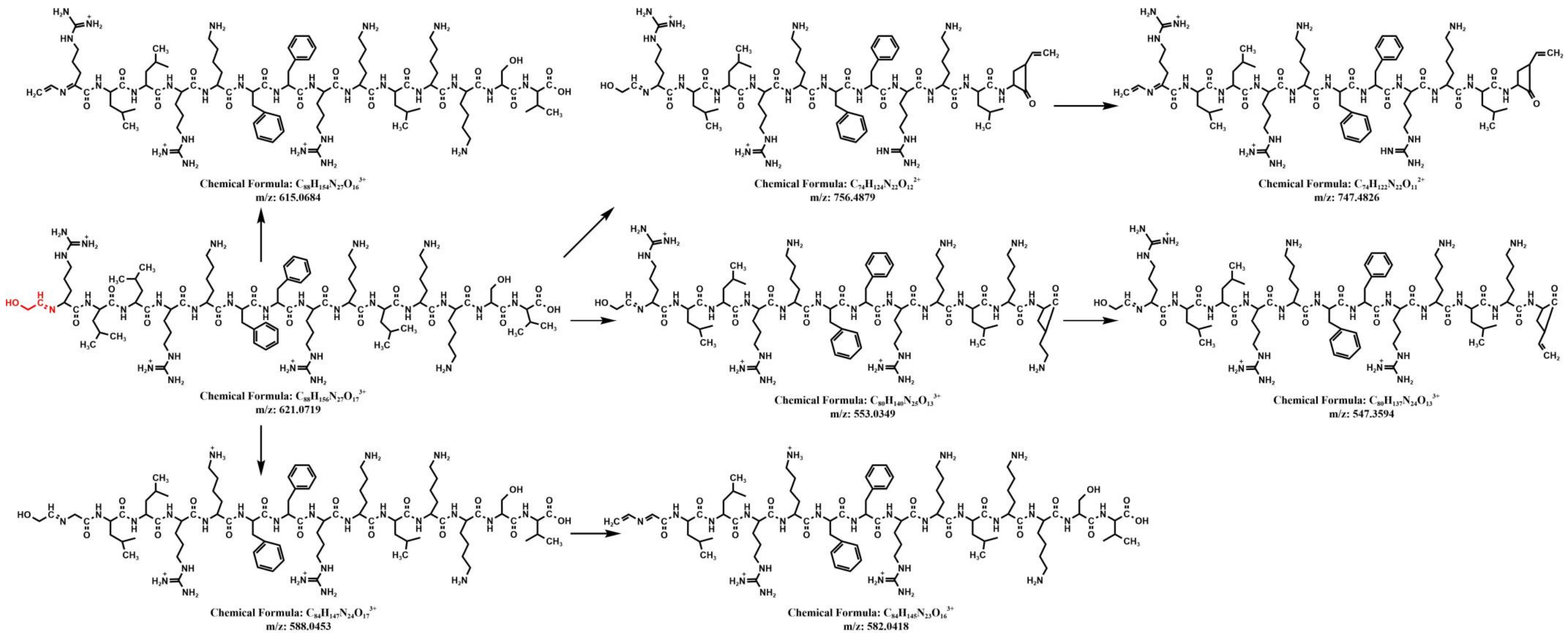
Chemical Formula: $C_{88}H_{154}N_{27}O_{16}^{3+}$
m/z: 615.0684
Chemical Formula: $C_{74}H_{124}N_{22}O_{12}^{2+}$
m/z: 756.4879
Chemical Formula: $C_{74}H_{122}N_{22}O_{11}^{2+}$
m/z: 747.4826
Chemical Formula: $C_{88}H_{156}N_{27}O_{17}^{3+}$
m/z: 621.0719
Chemical Formula: $C_{80}H_{140}N_{25}O_{13}^{3+}$
m/z: 553.0349
Chemical Formula: $C_{80}H_{137}N_{24}O_{13}^{3+}$
m/z: 547.3594
Chemical Formula: $C_{84}H_{147}N_{24}O_{17}^{3+}$
m/z: 588.0453
Chemical Formula: $C_{84}H_{145}N_{23}O_{16}^{3+}$
m/z: 582.0418

d

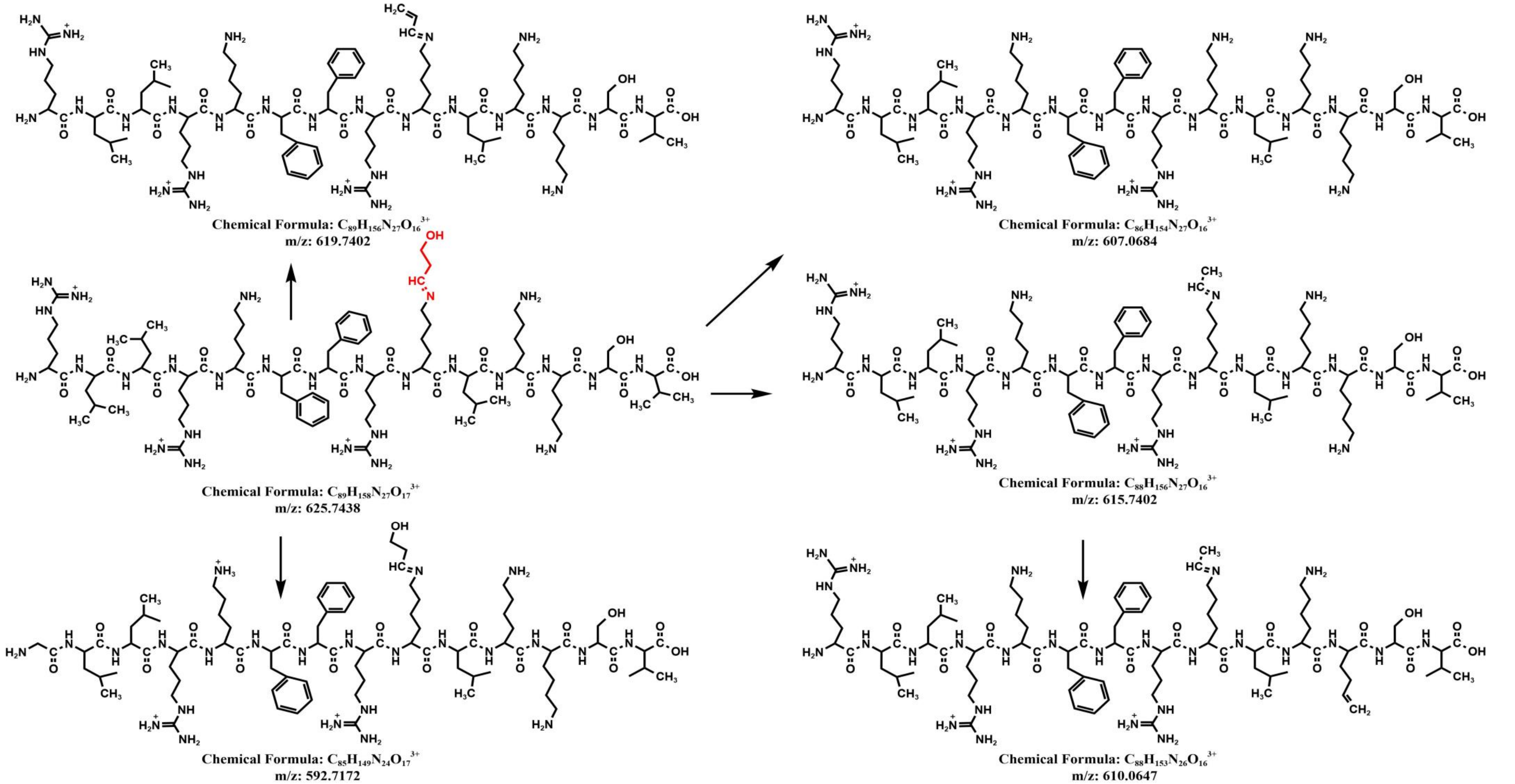
Chemical Formula: $C_{89}H_{156}N_{27}O_{16}^{3+}$
m/z: 619.7402
Chemical Formula: $C_{89}H_{158}N_{27}O_{17}^{3+}$
m/z: 625.7438
Chemical Formula: $C_{85}H_{149}N_{24}O_{17}^{3+}$
m/z: 592.7172
Chemical Formula: $C_{86}H_{154}N_{27}O_{16}^{3+}$
m/z: 607.0684
Chemical Formula: $C_{88}H_{156}N_{27}O_{16}^{3+}$
m/z: 615.7402
Chemical Formula: $C_{88}H_{153}N_{26}O_{16}^{3+}$
m/z: 610.0647

e

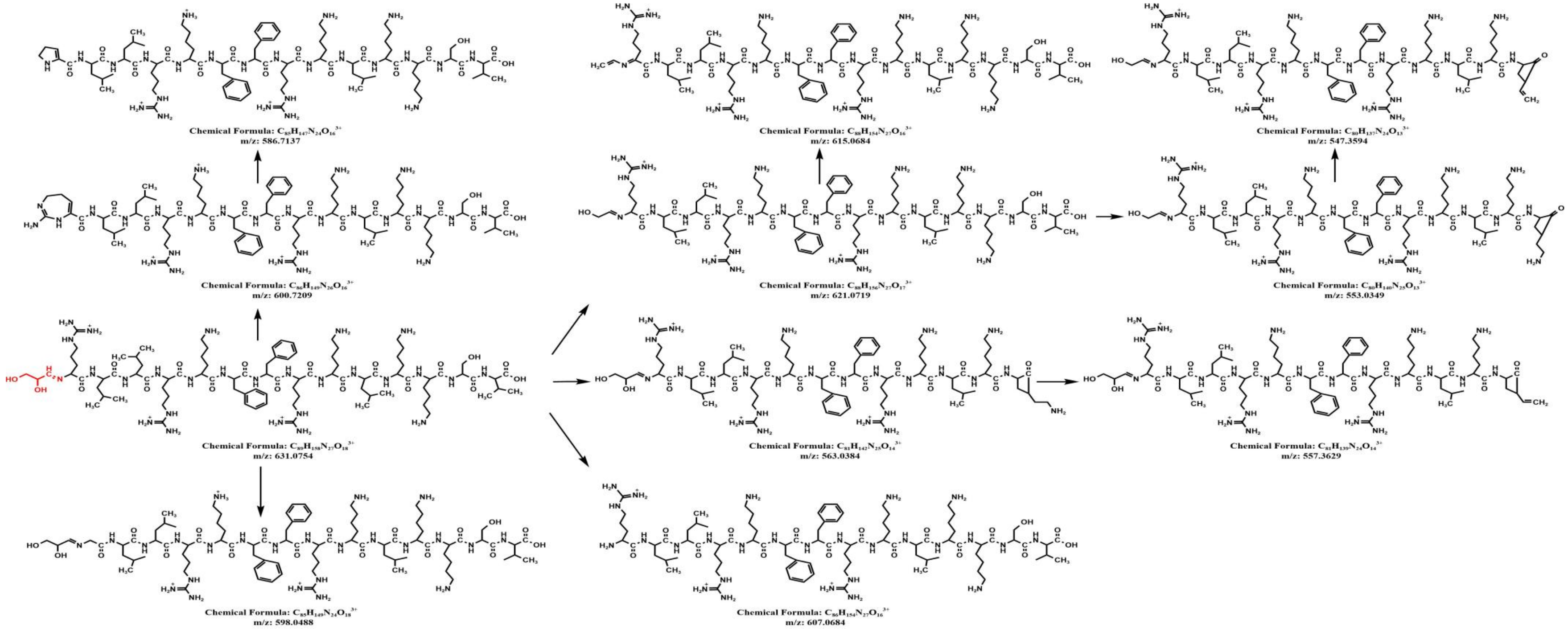

Figure S2. Plausible fragmentation pathways of (a) N-methylene-Cbf-14 (represented as impurity 2, and impurity 4, 6 were similar); (b) N-ethylidene-Cbf-14 (represented as impurity 1, and impurity 7 was similar); (c) N-(2-hydroxyethylidene)-Cbf-14 (represented as impurity 10, and impurity11 was similar); (d) N-(3-hydroxypropylidene)-Cbf-14 (impurity 8); (e) N-(2,3-dihydroxypropylidene)-Cbf-14 (represented as impurity 3, and impurity 5, 9 were similar).

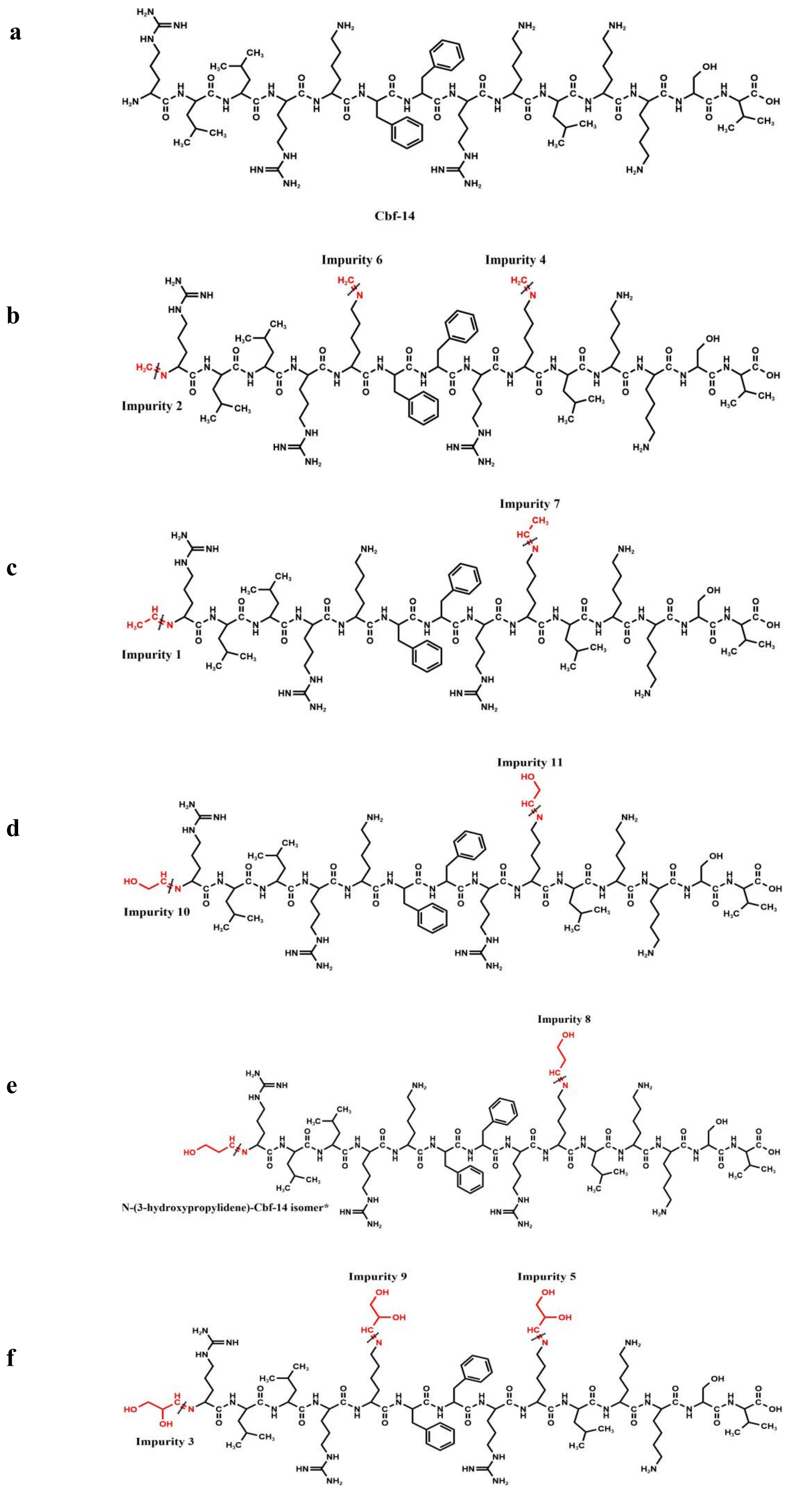
a
Cbf-14
b
Impurity 6
Impurity 4
Impurity 2
c
Impurity 7
Impurity 1
d
Impurity 11
Impurity 10
e
Impurity 8
N-(3-hydroxypropylidene)-Cbf-14 isomer*
f
Impurity 9
Impurity 5
Impurity 3

Figure S3. Deduced structures of compounds in this study. (a) Cbf-14; (b) N-methylene-Cbf-14 (impurity 2, 4, 6); (c) N-ethylidene-Cbf-14 (impurity 1, 7); (d) N-(2-hydroxyethylidene)-Cbf-14 (impurity 10, 11); (e) N-(3-hydroxypropylidene)--Cbf-14 (impurity 8); (f) N-(2,3-dihydroxypropylidene)-Cbf-14 (impurity 3, 5, 9).

* The impurity formed when Cbf-14 reacted with 3-hydroxypropanal (as shown in Fig 3e at t_R 24.8 min) was not detected in the gel.
